# Supplementary material for: Formation of a stable RNase Y-RicT (YaaT) complex requires RicA (YmcA) and RicF (YlbF)
Source: mBio. 2023 Aug 9;14(4):e01269-23. doi: 10.1128/mbio.01269-23 (PMC10470536; doi:10.1128/mbio.01269-23)
Supplement: Fig. S1 — Verification of the Ric-3FL constructs using Western blots. [file mbio.01269-23-s0001.pdf]

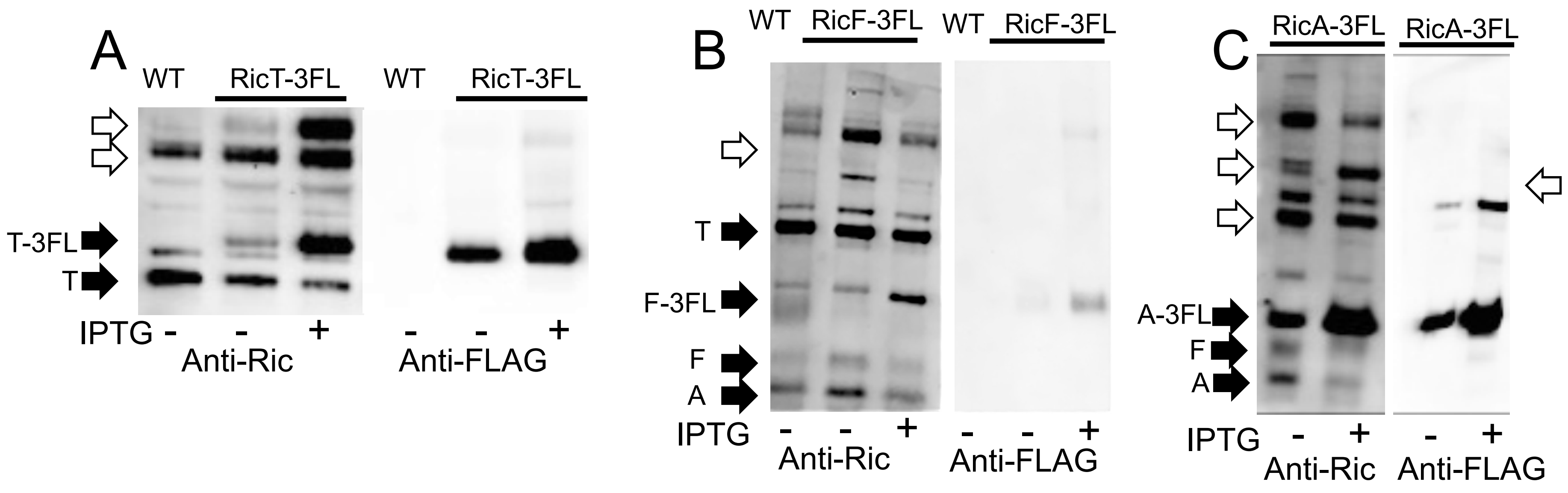

Fig. S1. Verification of the Ric-3FL constructs using Western blots. Panels A, B and C show that the RicT-3FL, RicF-3FL and RicA-3FL express the appropriate fusion proteins, detectable using both anti-Ric and anti-FLAG antisera and that the constructs are IPTG-inducible. These strains did not carry knockouts of the native *ric* genes and thus signals for the untagged proteins were detected.
