# Supplementary material for: Formation of a stable RNase Y-RicT (YaaT) complex requires RicA (YmcA) and RicF (YlbF)
Source: mBio. 2023 Aug 9;14(4):e01269-23. doi: 10.1128/mbio.01269-23 (PMC10470536; doi:10.1128/mbio.01269-23)
Supplement: Fig. S2 — Ric-3FL and Rny-3FL proteins support processing of the cggR gapA transcript. [file mbio.01269-23-s0002.pdf]

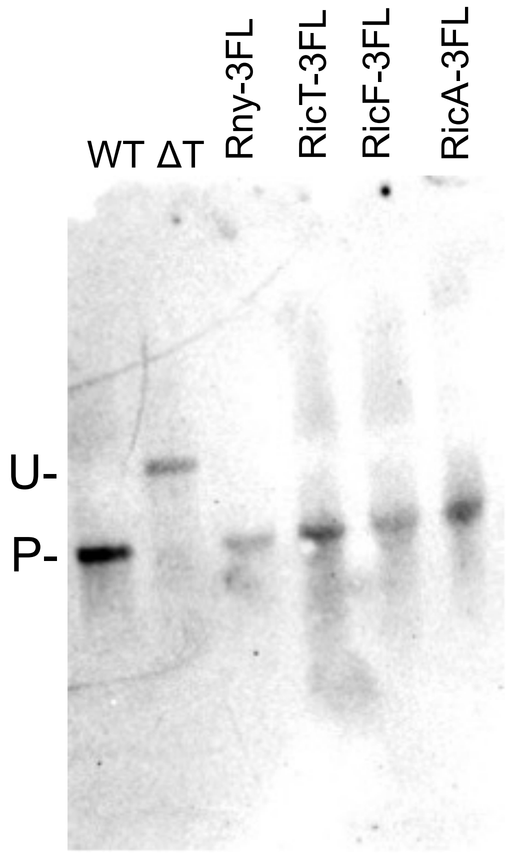

Fig. S2. Ric-3FL and Rny-3FL proteins support processing of the *cggR gapA* transcript. Northern blotting was carried out using a probe complementary to the coding strand of *gapA*. Each of the four fusion strains was the only source of RicA, RicF, RicT and Rny in the cells. U and P show the positions of the unprocessed and processed RNA species (~2.2 and ~1.2 kb, respectively). The *ricT* deletion and wild-type (IS75) control lanes show the positions of these two species, respectively.
