# Supplementary material for: Formation of a stable RNase Y-RicT (YaaT) complex requires RicA (YmcA) and RicF (YlbF)
Source: mBio. 2023 Aug 9;14(4):e01269-23. doi: 10.1128/mbio.01269-23 (PMC10470536; doi:10.1128/mbio.01269-23)
Supplement: Fig. S3 — Verification of anti-serum specificities and of the anti-FLAG magnetic beads. [file mbio.01269-23-s0003.pdf]

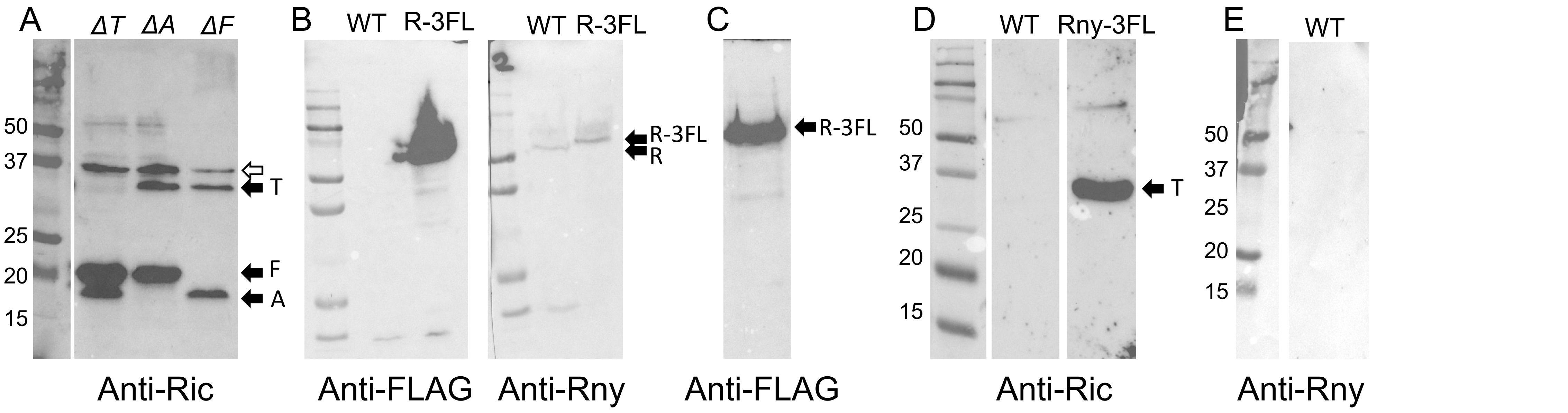

Fig. S3. Verification of anti-serum specificities and of the anti-FLAG magnetic beads. (A). The appropriate Ric proteins bands are missing from total lysates of each indicated deletion strain. A cross-reacting band is indicated with an empty arrow. (B) In a lysate of the Rny-3FL strain, the anti-FLAG and anti-Rny antisera reveal the fusion protein, but only the wild-type Rny signal is evident in a wild-type lysate. (C) An eluant fraction is shown from a pull-down experiment using an Rny-3FL lysate and anti-FLAG antiserum. The magnetic beads successfully pull down Rny-3FL. (D) Eluant fractions from pull-down experiment using wild-type and Rny-3FL lysates. The blots were developed with anti-Ric antiserum. RicT does not bind non-specifically to the beads. (E) No signal is detected in the eluant fraction from a mock pull-down experiment with a wild-type lysate, using anti-Rny antiserum. Rny does not bind non-specifically to the anti-FLAG magnetic beads. The Rny antiserum is validated in Fig. 2 panels B and C.
