# Supplementary material for: Formation of a stable RNase Y-RicT (YaaT) complex requires RicA (YmcA) and RicF (YlbF)
Source: mBio. 2023 Aug 9;14(4):e01269-23. doi: 10.1128/mbio.01269-23 (PMC10470536; doi:10.1128/mbio.01269-23)
Supplement: Fig. S4 — The three Ric-3FL constructs can pull one another down with anti-FLAG beads. [file mbio.01269-23-s0004.pdf]

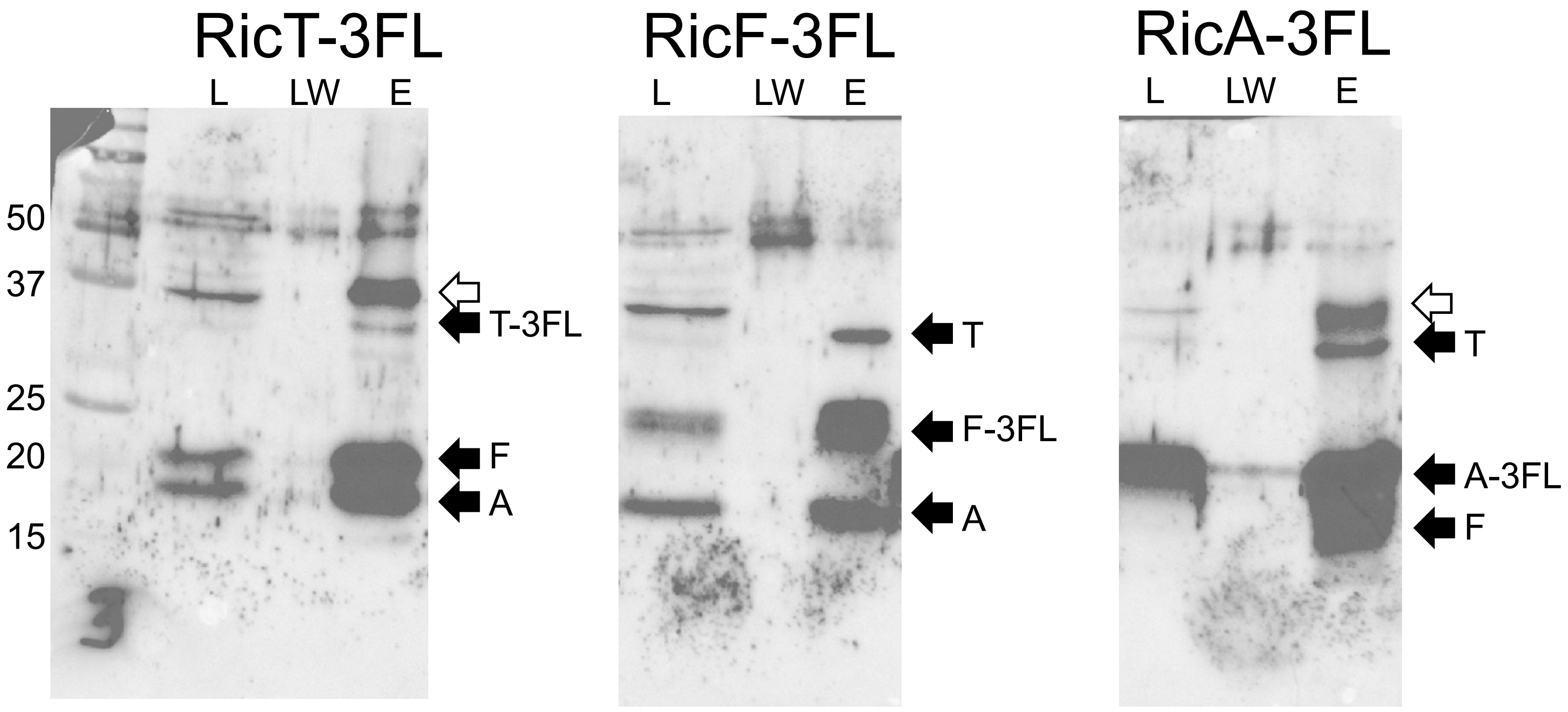

Fig. S4. The three Ric-3FL constructs can pull one another down with anti-FLAG beads. In all three panels the blots were developed with anti-Ric antiserum. The empty arrow shows a cross-reacting band.
