# Supplementary material for: Formation of a stable RNase Y-RicT (YaaT) complex requires RicA (YmcA) and RicF (YlbF)
Source: mBio. 2023 Aug 9;14(4):e01269-23. doi: 10.1128/mbio.01269-23 (PMC10470536; doi:10.1128/mbio.01269-23)
Supplement: Table S2 — Strains. [file mbio.01269-23-s0006.pdf]

Table S2

Strains

Strain number	Relevant genotype[a]	Source
IS75[a]	*his leu met*	Lab strain
BD9000	*rny-3FL (spc)*	This work
BD9170	P_{spac}-*ricT-3FL::thr (erm) ricT::kan*[b]	This work
BD9171	P_{spac}-*ricF-3FL::thr (erm) ricF:(kan*[b]	This work
BD9172	P_{spac}-*ricA-3FL::thr (erm) ric::kan*[b]	This work
BD9195	*rny-3FL (spc) ΔricA*[c]	This work
BD9196	*rny-3FL (spc) ΔricF*[c]	This work
BD9210	*rny-3FL (spc) ricT C161S*	This work
BD9211	*rny-3FL (spc) ricT C198S*	This work
BD9212	*rny-3FL (spc) ricT C167S*	This work
BD9217	P_{spac}-*ricT-3FL::thr (erm) rny::kan*[b]	This work
B9218	P_{spac}-*ricT-3FL::thr (erm) rny::kan*[b] *ricA::spc*[d]	This work

[a]All strains were constructed in the IS75 background.

[b]These knockouts were obtained from the Bacillus Genetic Stock Center and were constructed by Koo et al (36).

[c]These deletions are markerless. Cassettes were removed using pDR244 (36).

[d]A gift from the late A. A. Neyfakh.
