# Supplementary material for: Formation of a stable RNase Y-RicT (YaaT) complex requires RicA (YmcA) and RicF (YlbF)
Source: mBio. 2023 Aug 9;14(4):e01269-23. doi: 10.1128/mbio.01269-23 (PMC10470536; doi:10.1128/mbio.01269-23)
Supplement: Table S3 — Oligonucleotides. [file mbio.01269-23-s0007.pdf]

Table S3

Oligonucleotides

| **Name** | **Sequence**[a] |
|---|---|
| Rny-F | GTCATC**GAATTC**TTCGTAAAACCATTGCCG |
| Rny-R | GTCATC**GGATCC**TTTTGCATACTCTACGGC |
| RicT-F1 | CGAG**GGTACC**CTTGGTGTTAATGTTGCAGG |
| RicT-R1 | GTATCCGCACAAACCACAGATAGCGCT**ATCG**[b] |
| RicT-F2 | CCAAAAAACTGCTGCCTTC**GGATCC**GATATCCTAACAGCACAAGAG |
| RicT-R2 | GGGCTTTTTTCCATGCAAGCTAATTCGGATCC**AAGCTT**ATCGAATTCGATA |
| **RicA-F** | TGATCTAGAGTCGAG**GGTACC**GAAAGGAGAAAATATGACATG |
| **RicA-R** | CAAATAACAGCTGTTCTCTC**ATCGAT**CGGCCGGATTATAAGGAT |
| **RicF-F** | TGATCTAGAGTCGAG**GGTACC**CTGGAGGTGCATGTTATG |
| **RicF-F** | CTGCGGATGTAAAGTGTCC**ATCGAT**CGGCCGGATTATAAGGAT |

[a]Relevant restriction sites are in bold face.

[b]This incomplete *Cla*I site is completed by the Gibson Assembly step.
